# A preclinical intraneuronal stage of autophagy‐lysosomal dysfunction, amyloidosis, and neuron death yields senile plaques in human late‐onset Alzheimer’s Disease

**DOI:** 10.1002/alz.095591

**Published:** 2025-01-09

**Authors:** JU‐HYUN LEE, Philip Stavrides, Sandipkumar Darji, Martin J. Berg, Chris N. Goulbourne, Panaiyur S. Mohan, Cynthia Bleiwas, James Peddy, Eric B. Dammer, Nicholas T Seyfried, Ralph A. Nixon

**Affiliations:** ^1^ Nathan Kline Institute for Psychiatric Research, Orangeburg, NY USA; ^2^ New York University Langone Health, New York, NY USA; ^3^ Emory University School of Medicine, Atlanta, GA USA

## Abstract

**Background:**

Autophagy‐lysosomal pathway (ALP) efficiency declines Alzheimer’s disease (AD). In AD mouse models expressing a fluorescent autophagy and pH probe, autolysosomes pH elevation, resulting from deficient v‐ATPase activity, causes autophagy substrates, including Aβ and APP‐βCTF, to build up selectively within autolysosomes before extracellular amyloid deposits. In the most compromised but still intact neurons, massive numbers of Aβ‐positive autolysosomes pack into huge petal‐like blebs bulging out from the perikaryal membrane (PANTHOS). They additionally fuse with endoplasmic reticulum during its disrupted autophagic turnover causing plaque‐like fibrillar β‐amyloid to accumulate in tubular membrane networks. As the neuron dies, the extracellular β‐amyloid plaque left behind becomes the major source of senile plaques in early disease, supporting their “inside‐out” origin. Here, we show that the same pathological sequence evolves similarly in late‐onset AD human brain.

**Method:**

Our published curated autophagy pipelines were used in a deeper interrogation of earlier unbiased WTA analyses of ROSMAP/Banner transcriptomic and proteomic datasets and additional transcriptomic profiles of individual brain cell types from postmortem AD human brains. Complementary analyses included multiplex immunohistochemistry, biochemical and ultrastructural approaches.

**Result:**

Omic analyses of ALP revealed in AD brains a selective down‐regulation of genes and proteins involved in lysosomal efficiency such as v‐ATPase complex, lysosomal acidification, and regulation of lysosomal environments, relative to upstream autophagy pathway components. Notably, excitatory neurons, among brain cell types, exhibited similar lysosomal/acidification findings in preclinical stage AD. At the exceptionally early “intraneuronal” pre‐plaque stage of AD pathology, susceptible neuronal populations develop progressive ALP pathology leading to intraneuronal β‐amyloid aggregate formation, similar to the PANTHOS pattern seen in mice. Close temporal and 1:1 spatial correlation between PANTHOS, intracellular Aβ accumulation, and plaque formation in early Braak‐stage strongly suggests a significant association between PANTHOS and the progression of amyloid pathology in late‐onset AD.

**Conclusion:**

Beginning at a preclinical stage, vulnerable neuron populations in human sporadic AD develop the same PANTHOS pattern of ALP dysfunction and neuronal cell death as observed uniquely in mouse models of AD, which arises from APP‐dependent lysosomal acidification deficiency. ALP failure and PANTHOS development results in early selective neuronal death that initiates emergence of amyloid plaques. Grants P01AG017617, R01AG062376.

